# Immune reconstitution syndrome presenting as probable AIDS-related lymphoma: a case report

**DOI:** 10.1186/1742-6405-8-34

**Published:** 2011-09-28

**Authors:** Bo K Mortensen, Susanne D Nielsen, Charlotte B Christensen, Jannik Helweg-Larsen

**Affiliations:** 1 Department of Infectious Diseases, Rigshospitalet, Copenhagen, Denmark; 2 Department of Hematology, Rigshospitalet, Copenhagen, Denmark; 3 Department of PET/CT imaging, Rigshospitalet, Copenhagen Denmark

**Keywords:** Immune reconstitution inflammatory syndrome, AIDS related lymphoma, Cart, HIV, 18F-FDG PET/CT

## Abstract

We report an unusual case of HIV-related immune reconstitution inflammatory syndrome, presenting as suspected AIDS-related lymphoma. Symptoms, initial investigations including fine-needle biopsy and 18F-FDG PET/CT scan were highly compatible with high grade AIDS-related lymphoma, however subsequently IRIS was diagnosed. We discuss pitfalls in the interpretation of diagnostic results in ARL versus IRIS.

## Background

Immune inflammatory reconstitution syndrome (IRIS) in HIV patients after initiation of combination antiretroviral therapy (cART) has been increasingly recognized. IRIS is defined as a paradoxical worsening or unmasking of infections, autoimmune diseases or tumours after initiating of cART, and is usually seen when the initial CD4 count is low. The pathological basis is thought to be recovery of the immune system, and IRIS is often described to occur in patients infected with mycobacteria. The diagnosis of IRIS is often challenging as differential diagnosis is complex, including opportunistic infection, drug failure/toxicity or malignancy. The most common symptoms seen in IRIS are fever, lymphadenopathy and pulmonary symptoms [1] which resemble symptoms seen in patients with AIDS related lymphoma (ARL). In the literature, cases of IRIS, mimicking relapse of ARL have been described [2]. In addition, high rate of ARL has recently been reported shortly after initiation of newer-class antiretrovirals [3] which makes the distinction between ARL and IRIS important. As seen in the present case report, the distinction between ARL and IRIS may cause major difficulties.

## Case report

A 37-year homosexual male was admitted to a Gastroenterology Clinic because of six weeks of non-bloody diarrhea, abdominal pain and marked weight loss. Findings at sigmoideoscopy suggested Crohns Disease. After one month of steroid treatment most symptoms had subsided with colonoscopy and x-ray of the small intestine then demonstrating only mild colitis without involvement of the terminal ileum. In the following weeks, steroids were completely tapered; however HIV-testing was not performed.

Four month after the first admission, diarrhea and weightloss recurred. At readmission to hospital, moderate anemia and thrombocytopenia were found. An abdominal ultrasound demonstrated splenomegaly. Testing for HIV was positive with signs of severe immunodefiency with a CD4 count of 32 cells/microliter.

Combination antiretroviral treatment (cART) treatment was initiated. However, during the next five weeks the patients overall condition slowly deteriorated with severe weight loss, fever and night sweats. At hospital admission at five weeks of cART, the patient was now severely prostrated with anemia and high LDH. CT scan revealed bulky mediastinal and retroperitoneal glands, the largest measuring 4.5 × 6 × 9 cm. ARL was suspected.

**Figure 1:**
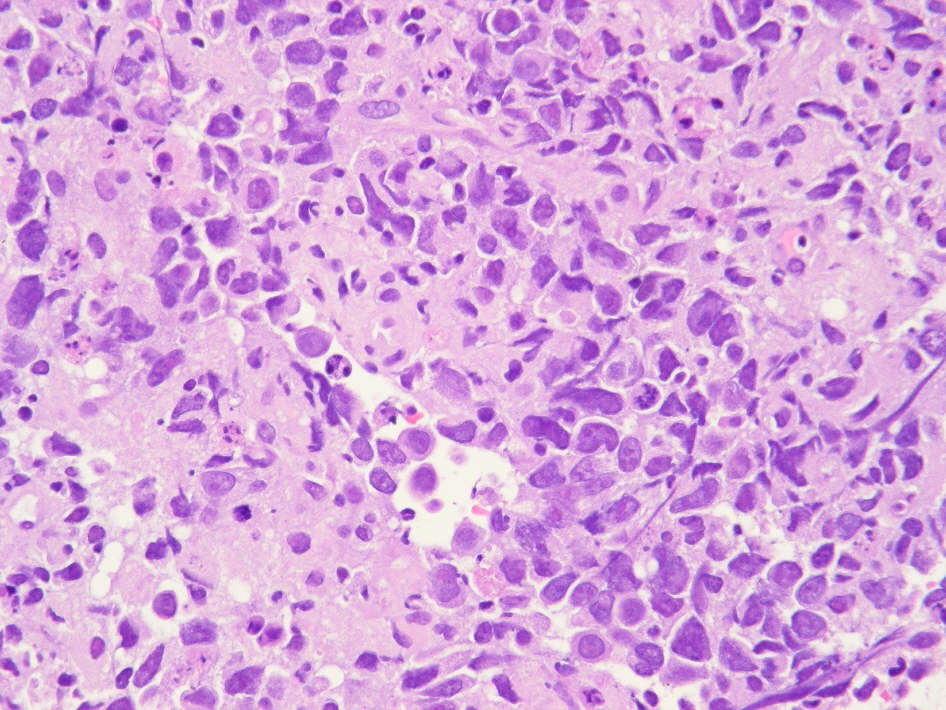
**Initial fine needle cytology from mediastinal gland suggestive of widespread tumor cell infiltration**.

An ultrasound-guided transesophagal needle aspiration cytology and biopsy of a mediastinal lymph node was performed. The cytology was suggestive of widespread tumor cell infiltration, Figure 1. In this biopsy, the supposed malignant cells contained multiple mitotic figures, with a rich amount of cytoplasma, large dark nuclei together with several apoptotic cells. Malignant cells were positive for CD30, EMA, Ki-67 and vimentin, and some were positive for MelanA and CD45. All tumor cells were negative for KL1, TTF-1, chromogranine, alfa feto protein and CD15. No acid-fast bacilli and no granulomas were seen. On the basis of these findings, malignant lymphoma, probably anaplastic large cell lymphoma, was strongly suspected. However a rebiopsy was recommended for definitive classification. One week after the first biopsy, and seven weeks after initation of cART, ultrasonic guided biopsy from the previously observed abdominal lymph nodes was attempted, but surprisingly enlarged lymph nods could not be demonstrated by ultrasound. Instead a lymph node was removed by mediastinoscopy.

**Figure 2:**
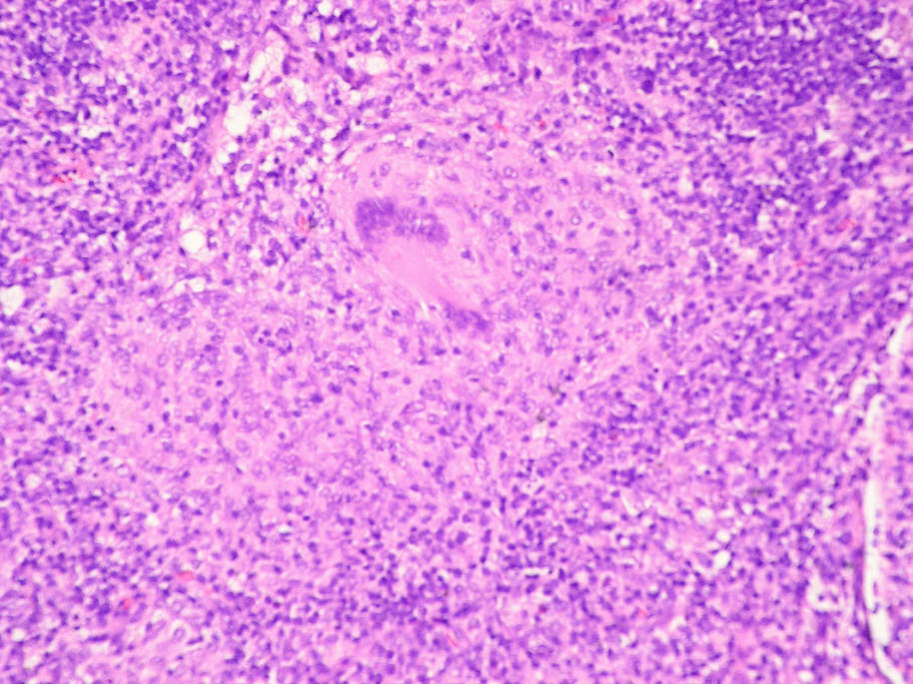
**Mediastinal gland showing granulomas with epitheloid cells and necrosis**.

Immediately after this biopsy, pre-treatment with a single dose of vincristine (1 mg) and 100 mg of prednisolone daily for one week was started according to the R-CHOP-14 protocol [2] due to the persistent severe B-symptoms and the high suspicion of lymphoma. However, no malignant cells were detected in the mediastinal lymph node removed. Instead granulomas with epitheloid cells and necrosis were observed, Figure 2. Intensive workup for mycobacterial disease was, however, negative. The biopsy was PCR negative for mycobacteria. Sputum, gastric lavage and peripheral blood were all culture and PCR negative for tuberculous and non-tuberculous mycobacteriae. In addition, interferon-gamma release assay testing for latent TB (Quantiferon Gold-TB test) was negative as well. An 18F-FDG PET/CT scan two weeks after the first CT-scan and eight weeks after initiation of cART now revealed metabolic active but significantly regressed lymph nodes in mediastinum and retroperitoneum. Because of these findings, the diagnosis of suspected ARL was reconsidered, and IRIS was suspected. Steroid treatment was slowly tapered.

**Figure 3:**
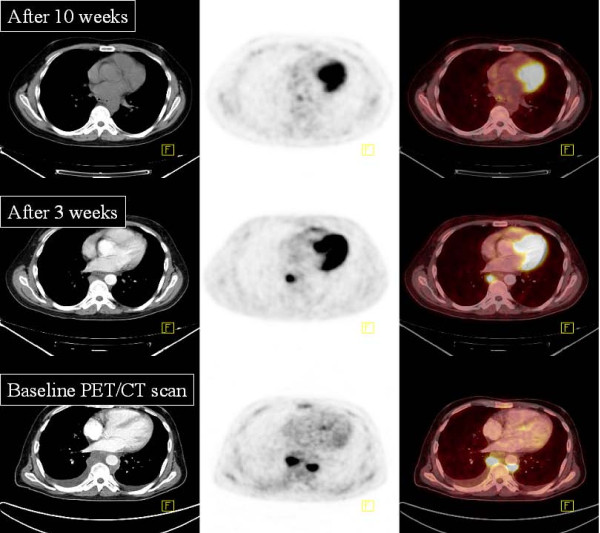
**18F-FDG- PET/CT findings**. Baseline: At presentation high metabolic activity in mediastinal mass. After 3 weeks: Both structural and metabolic regression. After 10 weeks: Complete structural and metabolic remission.

Repeated 18F-FDG PET/CT scan 11 weeks after initiation of cART showed both further structural and metabolic regression, Figure 3. At this time point, serum LDH had decreased, and the hemoglobin and platelet counts were almost normalized.

A final 18F-FDG PET/CT scan 18 weeks after initiation of cART revealed complete regression of all previous pathologic findings, at which point steroid treatment was discontinued. During continued antiretroviral therapy and two years of follow-up the patient has had a good immunologic recovery without signs of relapse.

## Discussion

The present case demonstrates pitfalls in interpretation of the standard techniques when diagnosing a patient with ARL versus IRIS.

At presentation, ARL was strongly suspected on the basis of initial biopsy findings compatible with high-grade lymphoma and PET/CT findings suggesting bulky, PET-positive malignancy.

Due to the high suspicion of ARL, pre-treatment with steroids was initiated, however a second biopsy only showed granulomas and inflammation, which were compatible with IRIS.

Patients with HIV and low CD4 counts presents a particular challenge in the interpretation of 18F-FDG PET/CT scans as a variety of infections and malignancies may cause increased FDG uptake [4]. In the present case, the pronounced lymph node enlargements and increased FDG-uptake was believed to be primarily compatible with aggressive ARL, however, as shown, the presence of HIV should always increase the index of suspicion that pathology other than lymphoma could be present.

The pathogenesis of IRIS remains unclear, but is believed to be mediated by the recovery of immune responses to pre-existing provoking antigens, usually opportunistic pathogens. The main risk factors for IRIS are severe CD4 lymphopenia and infection at cART initiation, which leads to a dysregulated T-cell response to antigen when severe immunosuppression reverses. In tuberculosis-associated IRIS, the exacerbation reflects restoration of antimycobacterial IFN-γ producing T-cells followed by a storm of pro-inflammatory Th1 cytokines which leads to lymph node enlargement and granuloma formation [5]. However, the finding of granulomatous inflammation is unspecific and has also been described in connection with IRIS caused by fungi or protozoa [6]. Noninfectious etiologies to IRIS have also been reported [7] in association with autoimmune manifestations such as as sarcoidosis and sarcoid-like pulmonary disorders [8,9]. In such cases, it is presumed that an autoimmune reaction triggered by self antigens leads to a Th1 respons followed by a granulomatous reaction [10].

Sarcoid-like granulomas have often been described in association with lymphomas, both in Hodgkins disease and in Non-Hodgkin lymphomas [11]. Similar to HIV-associated IRIS, it is believed that aberrant cytokine production in the tumor cells or other cells composing the tumor background may provoke a granulomatous reaction.

The exact cause of IRIS in this present case remains unknown. The most likely explanation for the observation of granulomas are infection with Mycobacteria or fungi [12]. However we believe that the clinical course and microbiological findings in our case were not directly compatible with IRIS caused by opportunistic infections since mycobacterial cultures and extensive investigation for infections were negative and recovery occurred in the absence of anti-infectious therapy.

The initial transesophagal biopsy revealing tumor cells was a needle biopsy but with limited material. Interpretation of fine needle aspiration cytology and biopsy is also a particular challenge within HIV-patients. Lowe and coworkers found, that a definitive diagnosis could not be made with fine needle aspiration cytology in 31% of HIV patients with lymphadenopathy and therefore an open lymph node biopsy was often needed. Although fine needle aspiration cytology is often inadequate for definitive diagnosis of lymphoma, it is unusual to misinterpret inflammation as compatible with malignancy [13].

In conclusion this case illustrates the pitfalls of standard techniques, including 18F-FDG PET/CT scan and microscopy of a biopsy in the setting of HIV. If an HIV positive patient with a low CD4 count develops signs of ARL shortly after the introduction of cART, clinicians must be aware that IRIS could be the cause. In such cases, definitive diagnostic lymph node excisional biopsy is mandatory.

## Consent

Written informed consent was obtained from the patient for publication of this case report and any accompanying images.

## Competing interests

The authors declare that they have no competing interests.

## Authors' contributions

All authors contributed to the manuscript. BM was responsible for the final manuscript, CC provided PET-CT images and interpretation. JH and SN were responsible for the primary management of the patient. JH has given the final approval of the version to be published. All authors have read and approved the final manuscript.
